# Effects of Including Misidentified Sharks in Life History Analyses: A Case Study on the Grey Reef Shark *Carcharhinus amblyrhynchos* from Papua New Guinea

**DOI:** 10.1371/journal.pone.0153116

**Published:** 2016-04-08

**Authors:** Jonathan J. Smart, Andrew Chin, Leontine Baje, Madeline E. Green, Sharon A. Appleyard, Andrew J. Tobin, Colin A. Simpfendorfer, William T. White

**Affiliations:** 1 Centre for Sustainable Tropical Fisheries and Aquaculture & College of Marine and Environmental Sciences, James Cook University, Townsville, Queensland, Australia; 2 National Fisheries Authority, National Capital District, Port Moresby, Papua New Guinea; 3 Institute for Marine and Antarctic Studies, University of Tasmania, Hobart, Australia; 4 CSIRO Oceans & Atmosphere, Hobart, Australia; 5 Australian National Fish Collection, CSIRO National Research Collections Australia, Hobart, Australia; Department of Agriculture and Water Resources, AUSTRALIA

## Abstract

Fisheries observer programs are used around the world to collect crucial information and samples that inform fisheries management. However, observer error may misidentify similar-looking shark species. This raises questions about the level of error that species misidentifications could introduce to estimates of species’ life history parameters. This study addressed these questions using the Grey Reef Shark *Carcharhinus amblyrhynchos* as a case study. Observer misidentification rates were quantified by validating species identifications using diagnostic photographs taken on board supplemented with DNA barcoding. Length-at-age and maturity ogive analyses were then estimated and compared with and without the misidentified individuals. Vertebrae were retained from a total of 155 sharks identified by observers as *C*. *amblyrhynchos*. However, 22 (14%) of these were sharks were misidentified by the observers and were subsequently re-identified based on photographs and/or DNA barcoding. Of the 22 individuals misidentified as *C*. *amblyrhynchos*, 16 (73%) were detected using photographs and a further 6 via genetic validation. If misidentified individuals had been included, substantial error would have been introduced to both the length-at-age and the maturity estimates. Thus validating the species identification, increased the accuracy of estimated life history parameters for *C*. *amblyrhynchos*. From the corrected sample a multi-model inference approach was used to estimate growth for *C*. *amblyrhynchos* using three candidate models. The model averaged length-at-age parameters for *C*. *amblyrhynchos* with the sexes combined were $\;{\bar{L}}_{\infty }$ = 159 cm TL and $\;{\bar{L}}_{0}$ = 72 cm TL. Females mature at a greater length (*l*_*50*_ = 136 cm TL) and older age (*A*_*50*_ = 9.1 years) than males (*l*_*50*_ = 123 cm TL; *A*_*50*_ = 5.9 years). The inclusion of techniques to reduce misidentification in observer programs will improve the results of life history studies and ultimately improve management through the use of more accurate data for assessments.

## Introduction

Life history information such as growth and maturity are fundamental prerequisites for many demographic and population dynamics models [1]. Without life history estimates, demographic assessments can be produced using life history theory, although the estimates will contain higher levels of uncertainty [2]. Producing accurate life history information is therefore crucial to inform fisheries management and conservation. However, in instances where available life history information has been inaccurate, population declines have occurred through incidental overfishing [3]. The production of accurate life history estimates or a quantifiable uncertainty around them is therefore imperative for sustainable fishing and effective population management.

The Grey Reef Shark *Carcharhinus amblyrhynchos* is a medium bodied whaler shark (Family Carcharhinidae) which is reef associated and has a Indo–West and Central Pacific distribution [4]. *Carcharhinus amblyrhynchos* are caught in tropical fisheries throughout their range [5, 6] and are often landed as incidental catch in some commercial fisheries [7, 8]. In Papua New Guinea (PNG) a dedicated shark long-line fishery existed until July 2014 which developed from the tuna fishery in the 1990s [9]. *Carcharhinus amblyrhynchos* was a common species caught in this fishery, where they comprised ~11% of the total catch [9]. Despite being susceptible to fisheries across much of its range, life history information for *C*. *amblyrhynchos* is only available from Australia [10, 11], with some limited data available from Hawaii [12, 13] and Indonesia [5]. However, as *C*. *amblyrhynchos* is caught in larger numbers in PNG, life history information is needed from the local population to form the basis of effective fisheries management and conservation.

Many elasmobranch life history studies have used observer programs as an effective source for collecting life history samples [14, 15, 16]. However, many tropical fisheries do not have operational observer programs and as a result many reef associated shark species are still data deficient with regards to life history information. Recent studies have started to fill these gaps by providing life history information for reef elasmobranchs through fishery independent sampling—where researchers conducted field work to collect the samples [10, 17, 18]. While these studies are valuable for species that cannot be sampled by other means, they add mortality to the population and are logistically disadvantaged as they cannot match the level of fishing effort that observer programs can sample. Observer programs therefore have several benefits for collecting life history samples including larger sample sizes, shorter sampling time frames, greater spread of samples across size ranges, and greater geographic coverage. The opportunistic use of observer programs to source life history samples can therefore have considerable benefits for species that have previously been difficult to sample.

While observer programs provide several benefits in collecting biological data, an important factor to consider is the accuracy of species identification. When collecting life history samples for sharks, many observer programs require observers to record basic biological information (species, length and sex), record the maturity status of an individual when possible, and remove a section of vertebrae for ageing. While this allows a great amount of information to be collected quickly without the need for storing large volumes of biological samples, only the observer witnesses the whole specimen. Therefore, an important assumption of observer data is that species identification is accurate. However, realistically some level of error is inherent in observer species identifications and only recently has this been quantified [19]. Genetic validation has shown that observer error can be substantial for carcharhinid sharks caught in multi-species fisheries in northern Australia [19]. In the northern Australian study, species misidentification occurred at different rates depending on a combination of factors such as species, sex and size [19]. The highest misidentification rates (~20%) occurred for *C*. *limbatus* and *C*. *tilstoni;* two species that are morphologically similar and known to hybridise [19, 20]. When using observer sourced samples, these findings raise questions about how often misidentified sharks are unintentionally included in life history analyses and the level of error this introduces into estimates.

Species validation is becoming increasingly feasible due to recent technological advances. Identifying species in the field can be complicated as closely examining morphological features such as dentition or fin morphology can be difficult in field conditions, and for cryptic or “look-alike” species. However, preserving entire specimens is often not possible for fisheries observers as sharks are typically processed at sea. Recent advances in digital camera technology are beginning to overcome this issue as many “all weather” rugged camera models are now available that survive exposure at sea and can store large numbers of images. This technology facilitates the post-cruise validation of species identifications using photographs taken by fisheries observers at sea. While digital cameras have great potential for species validation *in situ*, genetic analyses in the laboratory are increasingly being used for species identifications. DNA barcoding of the cytochrome c oxidase I (COI) mitochondrial (mtDNA) gene has become an important tool that can rapidly and accurately assist in species identification and can overcome issues such as unknown or poorly defined morphological characteristics that complicate accurate identification of individuals at sea [21]. Due to these advantages, the use of DNA barcoding is becoming increasingly common in fisheries science [21] and has already been used to validate species identifications for fisheries observer programs [19]. Both DNA barcoding and the post-fishing trip inspection of specimen photos provide an opportunity to determine what effects species misidentification might have on life history estimates and ultimately minimise them.

In order to determine the effects of species misidentification in life history analyses, a case study is presented using *C*. *amblyrhynchos* sampled from the PNG longline fishery. Two types of species validation techniques were used to identify the misidentification rate: 1) diagnostic photographs of the specimens taken on-board by the fisheries observers; and 2) DNA barcoding using the COI gene. This integrated approach of combining genetic and life history analyses allowed the effects of including misidentified individuals in life history studies to be explored.

## Methods

### Ethics Statement

Vertebrae from *Carcharhinus amblyrhynchos* were collected from commercial longline operations operating in Papua New Guinea by an observer placed on the vessels by the National Fisheries Authority (NFA), the governing fisheries authority in Papua New Guinea. No specific permits or approvals were required to collect samples from the sharks caught by the longliners. All sharks from which vertebrae were taken were to be retained by the fishing vessels as part of their quota.

### Sample collection

Samples were collected in May and June 2014 by observers on board longline vessels operating in the Bismarck and Solomon Seas. The vessels targeted shark species by setting their gear close to the surface while using a maximum of 1200 hooks per set for an average soak time of 8–10 hours [9]. Biological information was recorded for each landed individual including the total length (TL), sex and maturity stage. The TL of each individual was measured to the nearest 1 mm following [22]. A section of vertebrae consisting of about 4–6 centra were removed from the vertebral column below the first dorsal fin and stored frozen. Frozen vertebral sections were sorted at the NFA provincial office in Rabaul, East New Britain, and then sent to the laboratories at James Cook University (JCU) in Townsville. Tissue samples (approximately 150 mg) for DNA barcoding were later excised from the remaining muscle around the vertebrae or from the vertebral chord and preserved in 100% analytical-grade ethanol.

**Fig 1:**
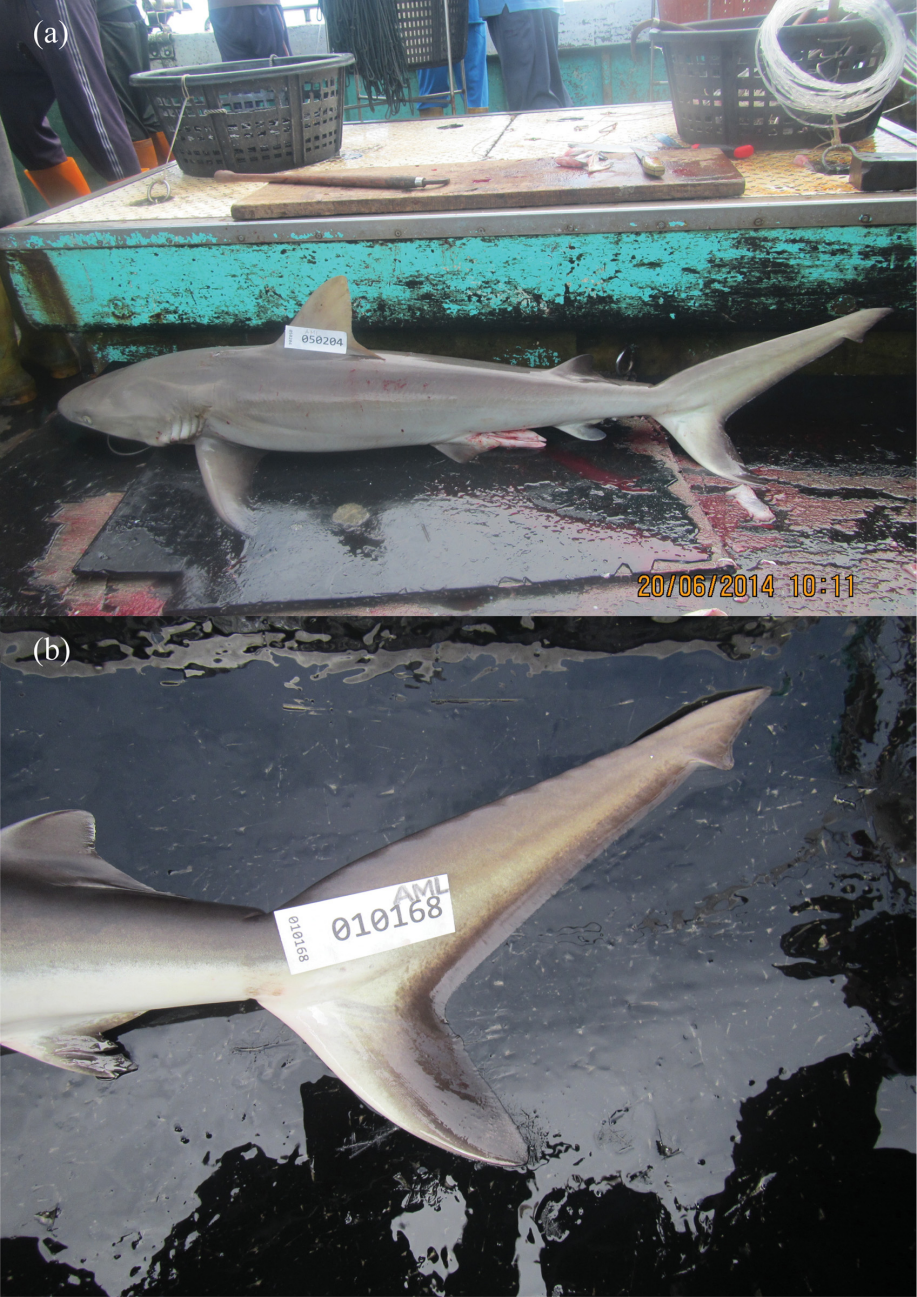
Diagnostic photographs of *C*. *amblyrhynchos* taken by the NFA observers on board long line vessels. These photographs include (a) a ventral view of the whole specimen and (b) a view of the caudal fin. *Carcharhinus amblyrhynchos* have a very distinctive, broad black posterior margin on the caudal fin.

While on board the vessels, the NFA observers photographed each individual before processing. These images usually consisted of a roughly lateral view of the shark (Fig 1a), but sometimes also included secondary images of other key diagnostic features (e.g. ventral view of the head, upper dentition, close-ups of fins). These images were later examined by WTW to verify on-board species identifications. Most *C*. *amblyrhynchos* identifications were easily confirmed from images of the caudal fin as this species has a distinctive black margin on the anterior edge of the fin (Fig 1b). In some instances, the image did not include the key diagnostic feature, i.e. the caudal fin, and thus accurate confirmation could not be made from the image.

### DNA barcoding of tissue samples

DNA from vertebral chord or muscle samples was extracted using the Wizard^®^ SV Genomic DNA Purification system (Promega, Australia) with starting material of approximately 0.25 g. Tissue extractions were undertaken using SV minicolumns following manufacturer’s instructions (including an overnight digestion at 55°C on an Eppendorf Thermomixer Comfort (Eppendorf, Australia) and the modifications of 400 *μ*g Proteinase K and DNA precipitated in 160 *μ*l nuclease free water. Each DNA sample was quantified on a Nanodrop 8000 UV-Vis Spectrophotometer (Thermo Scientific, USA).

Genetic species identification through barcoding of the COI mtDNA gene was undertaken using the universal Fish-BCL (5’-TCAACYAATCAYAAAGATATYGGCAC-3’) and Fish-BCH (5’-ACTTCYGGGTGRCCRAARAATCA-3’) primers [23]. PCRs were undertaken in 25 *μ*l using GoTaq^®^ Green Master Mix (Promega, USA), Bovine Serum Albumin (Promega, USA), 10 *μ*M primers and DNA quantities of between 8 and 20 ng. PCRs were performed in an Applied Biosystems GeneAmp^®^ PCR System 9700 (Life Technologies, Thermo Fisher Scientific, USA) with cycling conditions of 94°C × 3 min; 35 cycles of 94°C × 1 min, 50°C × 1 min 30sec, 72°C × 1 min; and a final extension of 72°C × 10 min. PCR products were visualised on 2.5% TAE agarose gels and fragments cleaned using an Agencourt AMPure XP PCR purification kit (Beckman Coulter, Australia) according to the manufacturer’s instructions.

PCR products were sequenced bi-directionally using the same primers as in the original PCR, BigDye^®^ Terminator v3.1 Cycle sequencing kit (Life Technologies) and an annealing stage of 50°C × 5 sec across 25 cycles. Cycle sequenced products were cleaned using the CleanSEQ kit (Beckman Coulter) according to the manufacturer’s instructions and run on an ABI 3130XL AutoDNA sequencer (Life Technologies).

Forward and reverse sequences (per gene fragment) were assembled into consensus sequences in Geneious^®^ R8.1.4 (Biomatters Ltd Auckland, New Zealand; http://www.geneious.com) using the de novo assembly tool. Consensus sequences were aligned within Geneious using the MUSCLE algorithm and sequence identity was confirmed by using the BLAST module in Geneious (http://blast.ncbi.nlm.nih.gov/Blast.cgi;Megablast) against GenBank (http://www.ncbi.nlm.nih.gov/genbank/). COI sequences were additionally compared to sequences publicly available in the Barcode of Life database (BOLD, http://www.boldsystems.org/index.php/IDS_OpenIdEngine).

### Vertebrae sectioning

Vertebrae processing and sectioning followed [24]. Vertebrae were defrosted and the remaining muscle tissue was removed using a scalpel while also separating individual centra and removing the haemal arches. Individual centra were then soaked in a 4% sodium hypochlorite solution for 30 min and rinsed under tap water to remove any remaining connective tissue. They were then placed in a drying oven at 60°C for 24 hours. A single centrum from each individual was sectioned using a low-speed circular saw with two diamond-tipped blades (Beuhler, Illinois, USA). These sections were made through the centrum focus at a thickness of 400 *μ*m. After sectioning, each centrum was mounted onto a microscope slide using Crystal Bond adhesive (SPI supplies, Pennsylvania, USA).

### Age determination

Individual ages were estimated by counting translucent and opaque bands in the *corpus calcareum* of the centra under transmitted light [24]. Annual growth deposition could not be validated in this study as the short sample collection period precluded validation techniques such as marginal increment analysis. However, age validation was previously attempted for *C*. *amblyrhynchos* from northern Australia using oxytetracycline mark recapture methods [10]. While these attempts were unsuccessful, individuals that were at liberty for 10 months displayed growth consistent with annual growth band deposition [10]. Based on this evidence and a strong body of literature which has validated the ages of several carcharhinid species [17, 25, 26] annual growth band deposition was assumed in this study.

Growth bands were counted by two independent readers to reduce growth read bias [24]. When counts differed between readers the samples were re-examined until a consensus age was reached. If no consensus age was reached, that centrum was removed from analysis. In order to simulate the scenario where misidentified individuals were incidentally included in growth analysis; individuals that were mistakenly identified as *C*. *amblyrhynchos* were also included in the samples. Neither reader had any knowledge of which individuals had been misidentified nor how many were included.

Inter-reader precision was conducted on the original counts of both readers for verified *C*. *amblyrhynchos* (i.e. misidentified individuals were not included). Percent agreement ± 1 year (*PA* ± 1 year) was calculated between growth band reads [24]. Bowker’s test of symmetry [27, 28], average percent error (*APE*) and Changs coefficient of variation (*CV*) [29] were used to test precision and whether the inter-reader variability was systematically biased. These statistics were calculated using the FSA package [30] in the ‘R’ program environment [31].

### Growth modelling

A contemporary framework using multi-model inference (*MMI*) was used to estimate growth following [32]. This approach incorporated *a priori* a set of three candidate models: the von Bertalanffy, Gompertz and logistic growth models (Table 1) and used Akaike's information criterion (*AIC*) to evaluate model performance and produce a set of weighted model average length-at-age estimates [32]. This approach provides more robust growth estimates than the *a priori* use of the von Bertalanffy growth function (VBGF) [33, 34]. All three models were parameterised to include a length-at-birth parameter (*L*_*0*_) and an asymptotic length parameter (*L*_*∞*_) as both of these can be compared directly between growth functions (Table 1).

**Table 1:** Model equations of the three *a priori* growth functions used to estimate length-at-age.

Growth function	Equation	Reference
von Bertalanffy growth function (VBGF)	$L_{t}=L_{0}+(L_{\infty }-L_{0})\left(1-\text{exp}\left(-kt\right)\right)$	[35]
Gompertz function	$L_{t}=L_{0}\text{exp}\left(\text{ln}\left(\frac{L_{\infty }}{L_{0}}\right)\left(1-\text{exp}\left(-gt\right)\right)\right)$	[36]
logistic function	$L_{t}=\;\frac{L_{\infty }L_{0}\left(\text{exp}\left(gt\right)\right)}{L_{\infty }+L_{0}\left(\text{exp}\left(gt\right)-1\right)}$	[37]

where *L*_*t*_ is length-at-age *t*, *L*_*0*_ is length-at-age 0, *L*_*∞*_ is asymptotic length, *k* and *g* are the different growth coefficients of the respective models (which are incomparable).

The best fit parameter estimates of all three growth models were estimated using the 'nls' function in the ‘R’ program environment [31]. The *AIC* values were also calculated in the ‘R’ program environment [31] and incorporated an additional bias correction algorithm (*AICc*) as the number of samples was less than 200 [38]. The *AICc* was calculated as:

$$AIC_{c}=AIC+\frac{2k\left(k+1\right)}{n-k-1}$$

where *AIC = nlog*(σ^2^) + 2*k*, *k* is the total number of parameters +1 for variance (σ^2^) and *n* is the sample size. The model with the lowest *AICc* value (*AIC*_*min*_) was the most appropriate. The remaining models were ranked using the *AIC* difference (Δ) which was calculated for each model (*i* = 1–3) as:

$$\Delta =AIC_{c}-AIC_{min}$$

Models with Δ of 0–2 had the highest support while models with Δ of 2–10 had considerably less support and models with Δ of >10 had little or no support [39]. *AIC* weights (*w*) represent the probability of choosing the correct model from the set of candidates and were calculated for each model (*i* = 1–3) as:

$$w_{i}=\frac{\text{exp}\left(-\frac{\Delta_{i}}{2}\right)}{{\sum^{\;}}_{j=1}^{3}\text{exp}\left(-\frac{\Delta_{j}}{2}\right)}$$

As *L*_*∞*_ was comparable between the three growth functions, a model averaged value was calculated for both parameters as:

$${\bar{L}}_{\infty }=\overset{3}{\underset{i=1}{\sum^{\;}}}w_{i}*L_{\infty ,i}$$

where ${\bar{L}}_{\infty }$ was the model averaged asymptotic length [33, 40]. The unconditional standard error of ${\bar{L}}_{\infty }$ was estimated as:

$$SE\left({\bar{L}}_{\infty }\right)=\;\overset{3}{\underset{i=1}{\sum^{\;}}}w_{i}*{\left(var\left(L_{\infty ,i}|g_{i}\right)+{\left(L_{\infty ,i}-{\bar{L}}_{\infty }\right)}^{2}\right)}^{1/2}$$

where *var*(*L*_*∞*,*i*_|*g*_*i*_) is the variance of parameter *L*_*∞*_ of model *g*_*i*_ [34]. As *L*_*0*_ is also comparable between model candidates, a model averaged value and unconditional standard error were also calculated for it using the same methods. The three growth completion parameters (*k*, *g*_*logistic*_ and *g*_*Gompertz*_) are incomparable between candidate models and therefore cannot be averaged between them [32].

A likelihood ratio test [41] was used to determine if growth should be estimated for separate or combined sexes. This test was only conducted on the verified *C*. *amblyrhynchos* individuals using the method outlined by [42] in Microsoft Excel. An assumption of likelihood ratios tests is that the age ranges of the data are equivalent. Therefore, as females younger than 3 years old were missing from the sample, the age range of the males was truncated to be equivalent for this analysis. Likelihood ratio tests cannot be conducted on model averages. Therefore, this analysis was conducted for all three candidate models to ensure that sexual dimorphism of growth was not model dependent and avoid a type II error.

Growth analyses were carried out on two data sets: 1) with all the individuals identified as *C*. *amblyrhynchos* in the field and 2) with individuals misidentified as *C*. *amblyrhynchos* removed. A likelihood ratio test [41] was used to statistically test for coincident curves between the two data sets.

### Maturity estimation

The maturity of each individual was staged on board using an index modified from [43] (Table 2). Male maturity stages were based on clasper condition (C = 1–3) and female maturity stages were based on uteri condition (U = 1–5) (Table 2). Maturity stage data was converted to a binary maturity category (immature = 0 and mature = 1) for statistical analysis. Estimates of length-at-maturity were produced for males and females using a logistic regression model [43]:

$$P\left(l\right)=P_{max}{\left(1+e^{-\text{ln}\left(19\right)(\frac{l-l_{50}}{l_{95}-l_{50}}}\right)}^{-1}$$

where *P*(*l*) is the proportion of the population mature at TL, *l* and *P*_*max*_ is the maximum proportion of mature individuals. The lengths that 50% and 95% of the population were mature (*l*_*50*_ and *l*_*95*_) were estimated using a generalised linear model (*GLM*) with a binomial error structure and a logit-link function in the ‘R’ program environment [31]. Estimates of age-at-maturity (*A*_*50*_ and *A*_*95*_) were estimated using the same methods. *l*_*50*_ and *A*_*50*_ were used as metrics to describe the approximate length and age at maturity for the population.

**Table 2:** Indices for staging maturity condition. Adapted from [43]Organ.

	Index	Description	Binary maturity condition
Female Uterus	U = 1	Uniformly thin tubular structure. Ovaries small and without yolked ova	Immature
	U = 2	Thin, tubular structure which is partly enlarged posteriorly. Small yolked ova developing	Immature
	U = 3	Uniformly enlarged tubular structure. Yolked ova developed	Mature
	U = 4	*In utero* eggs or embryos macroscopically visible	Mature
	U = 5	Post-partum—enlarged tubular structure distended	Mature
Male Clasper	C = 1	Not calcified; pliable with no calcification	Immature
	C = 2	Partly calcified	Immature
	C = 3	Rigid and fully calcified	Mature

Maturity estimates were also estimated twice: 1) with all the individuals identified as *C*. *amblyrhynchos* in the field and 2) with individuals misidentified as *C*. *amblyrhynchos* removed. A statistical difference between two sets of population maturity estimates was tested for using a likelihood ratio test with a *χ*^*2*^ distribution using the ‘drop1’ function in the ‘R’ program environment [31].

## Results

### Effects of species misidentification on life history estimates

A total of 155 sharks were originally identified as *C*. *amblyrhynchos* by the on-board fisheries observers. However, 22 of these individuals (14.2%) were subsequently found to be misidentified and were not *C*. *amblyrhynchos*. Sixteen of these identification errors (72.2%) were originally detected by examining the photographs taken by the observers. DNA barcoding corroborated these corrections and also detected an additional six misidentified individuals (Table 3). Three of the misidentified individuals were larger than the typical length range for *C*. *amblyrhynchos* (*c*.190cm TL) [11]; these larger individuals were detected from the observer photographs (Table 3). The species that had been incorrectly identified as *C*. *amblyrhynchos* were the bull shark (*C*. *leucas*), common blacktip shark (*C*. *limbatus*) and silky shark (*C*. *falciformis*).

**Table 3:** Individuals misidentified as *C*. *amblyrhynchos* by on-board observers.

Corrected species ID	Total Length (cm)	Age (Vertebral growth band count)	Detected via photograph	Detected via DNA barcoding
*Carcharhinus lecuas*	284	21	Yes	Yes
*Carcharhinus limbatus*	145	7	Yes	Yes
*Carcharhinus falciformis*	90	1	No	Yes
*Carcharhinus falciformis*	92	1	Yes	Yes
*Carcharhinus falciformis*	95	1	Yes	Yes
*Carcharhinus falciformis*	95	2	No	Yes
*Carcharhinus falciformis*	108	5	Yes	Yes
*Carcharhinus falciformis*	112	5	No	Yes
*Carcharhinus falciformis*	112	4	Yes	Yes
*Carcharhinus falciformis*	121	6	Yes	Yes
*Carcharhinus falciformis*	123	4	No	Yes
*Carcharhinus falciformis*	124	6	Yes	Yes
*Carcharhinus falciformis*	127	7	Yes	Yes
*Carcharhinus falciformis*	127	8	Yes	Yes
*Carcharhinus falciformis*	137	9	Yes	Yes
*Carcharhinus falciformis*	146	9	Yes	Yes
*Carcharhinus falciformis*	149	7	Yes	Yes
*Carcharhinus falciformis*	150	11	Yes	Yes
*Carcharhinus falciformis*	170	8	No	Yes
*Carcharhinus falciformis*	174	5	No	Yes
*Carcharhinus falciformis*	192	13	Yes	Yes
*Carcharhinus falciformis*	230	13	Yes	Yes

**Fig 2:**
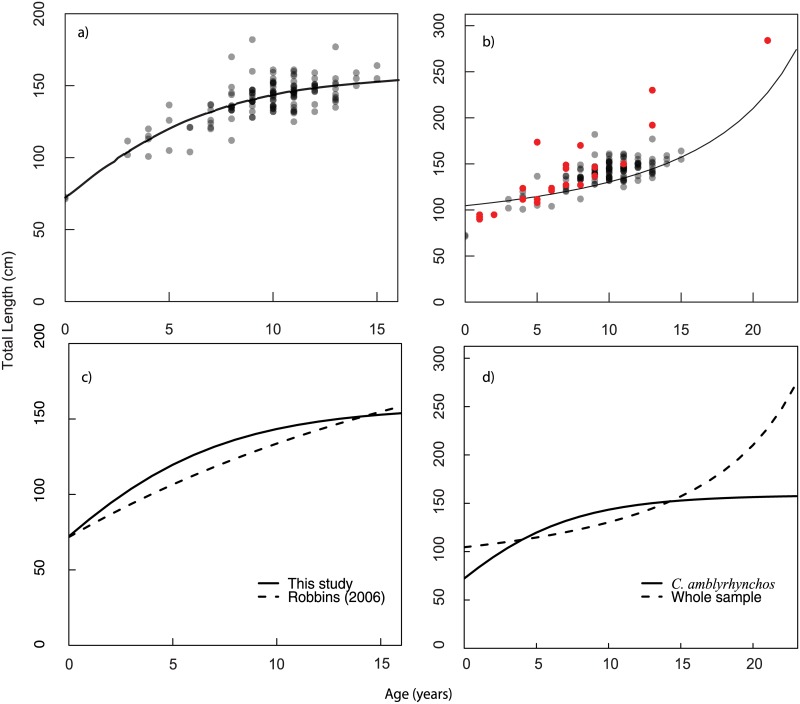
Length-at-age curves for: a) *C*. *amblyrhynchos*, b) *C*. *amblyrhynchos* (grey points) with misidentified individuals (red points) included, c) a comparison between *C*. *amblyrhynchos* from PNG (solid line) and northern Australia [10] (dashed line), and d) comparison of curves for *C*. *amblyrhynchos* (solid line) and *C*. *amblyrhynchos* with misidentified individuals included (dashed line). The species of the misidentifications are given in Table 3. All curves were fitted using the model averages of the *MMI* results except for the results from [10] which are the respective VBGF length-at-age estimates.

Likelihood ratio tests determined that the misidentified individuals produced a significantly different growth curve to *C*. *amblyrhynchos* when they were not removed (VBGF [*df* = 3, *χ*^*2*^ = 20.19, *p* = < 0.0001]; logistic function [*df* = 3, *χ*^*2*^ = 28.92, *p* = < 0.0001]; Gompertz function [*df* = 3, *χ*^*2*^ = 27.80, *p* = < 0.0001]). The *L*_*0*_ and *L*_*∞*_ parameter estimates did not resemble empirical length-at-birth or maximum length values and were extremely inflated (Fig 2b). The inclusion of misidentified individuals produced an ${\bar{L}}_{0}$ estimate of 105 cm TL which is well outside of the length-at-birth range of *C*. *amblyrhynchos* (63–72 cm TL) [11]. However, the greatest amount of error was introduced to the older age ranges of the growth curve (Fig 2b and 2d). The ${\bar{L}}_{\infty }$ estimate with the misidentified individuals included was 5640000 cm TL; a nonsensical value which demonstrated the inability of the model to include anomalous data produced by misidentification. This value was produced as the data was best fit by models that indicated growth increased continuously and therefore did not asymptote (Fig 2b and 2d). Subsequently all of the growth completion parameters (*k*, *g*_*logistic*_ and *g*_*Gompertz*_) were extremely low (Table 4). This growth trajectory occurred due to the inclusion of two individuals (230 and 284 cm TL) that were far larger than any of the verified *C*. *amblyrhynchos* individuals included in this study (Table 3).

**Table 4:** Summary of model parameters and *AIC*_*c*_ results for the observed length-at-age for *C*. *amblyrhynchos* and *C*. *amblyrhynchos* with misidentified individuals still included.

Model	*n*	AIC_*C*_	Δ	*w* (%)	*L*_∞_ (± *SE*)	*L*_*0*_ (± *SE*)	*k* (± *SE*)		*g*_*Gompertz*_ (± *SE*)	*g*_*logistic*_ (± *SE*)	*RSE*
*Carcharhinus amblyrhynch*os and misidentified individuals											
VBGF	155	1288.55	5.02	0.07	1.04e+4 (± 4.87e+5)	104 (± 5.69)	5.32e+4 (± 4.87e+5)		-	-	15.2
Logistic	155	1283.53	0.00	0.93	6.10e+6 (± 1.29e+11)	105 (± 4.37)	-		-	0.04 (± 0.02)	14.95
Gompertz	155	1545.85	262.33	0.00	1.27e+5 (± 9.41e+6)	105 (± 10.97)	-		5.93e+3 (± 0.06)	-	34.85
Model average	155	-	-	-	5.64e+6 (± 1.2e+11)	105 (± 4.45)	-		-	-	-
*Carcharhinus amblyrhynchos*											
VBGF	133	1000.52	0.32	0.30	163 (± 6.27)	71 (± 6.46)	0.15 (± 0.03)		-	-	9.92
Logistic	133	1000.20	0.00	0.35	156 (± 3.77)	73 (± 5.81)	-	-	-	0.26 (± 0.04)	9.91
Gompertz	133	1000.22	0.02	0.35	158 (± 4.65)	72 (± 6.14)	-	-	0.21 (± 0.03)	-	9.91
Model average	133	-	-	-	159 (± 5.62)	72 (± 6.20)	-	-	-	-	-

n is the sample size, *AIC*_*C*_ is the small-sample bias adjusted form of Akaike's Information Criteria, Δ is the difference in *AIC*_*C*_ values between models, *w* (%) are the *AIC*_*C*_ weights, *L*_*∞*_ is asymptotic length parameter in cm, *L*_*0*_ is the length-at-birth parameter in cm, *k* is the growth completion parameter in yr-1 for the VBGF, g is the growth parameter for Logistic and Gompertz functions (but is incomparable between the two), *SE* is the standard error of the adjacent parameter and *RSE* is the residual standard error of the model.

**Fig 3:**
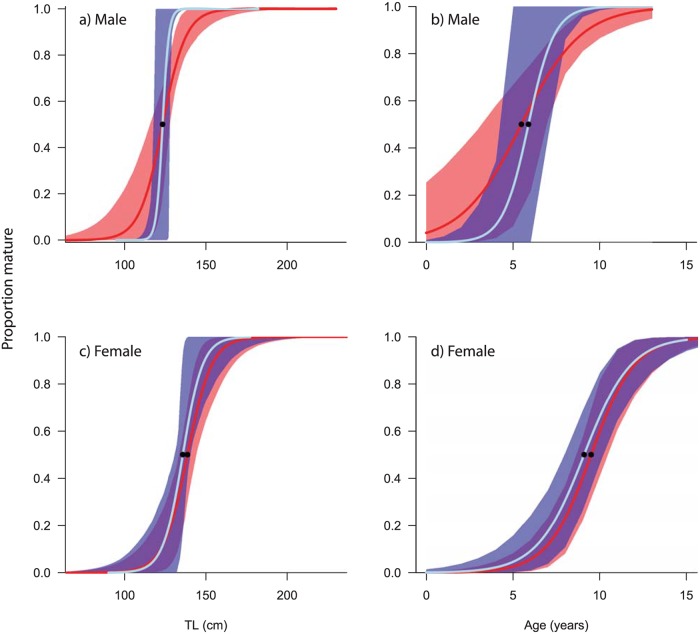
Length- and age-at maturity ogives for: (a, b) male and (c, d) female *C*. *amblyrhynchos* (light blue line) with 95% confidence intervals (blue area). The maturity ogives for *C*. *amblyrhynchos* when misidentified individuals were included with 95% confidence intervals are shown by the red line and red area respectively for comparison.

The maturity estimates were less affected than the growth estimates when misidentified individuals were included (Fig 3). Likelihood ratio tests determined that failing to remove misidentified individuals altered the maturity ogives for males (Length [*df* = 1, *χ*^*2*^ = 7.66, *p* = 0.005] and age [*df* = 1, *χ*^*2*^ = 4.03, *p* = 0.045]) but not for females (Length [*df* = 1, *χ*^*2*^ = 0.26, *p* = 0.61]; age [*df* = 1, *χ*^*2*^ = 0.03, *p* = 0.85]). However, the *l*_*50*_ and *A*_*50*_ estimates for males with misidentified individuals included were 123.3cm TL (*SE* = 3.12) and 5.5 years (*SE* = 0.85) respectively which were only marginally different to confirmed *C*. *amblyrhynchos*. The *l*_*50*_ and *A*_*50*_ estimates for females when misidentified individuals were included were 138.6 cm TL (*SE =* 2.96) and 9.5 years (*SE* = 0.52) respectively. Despite there being no significant difference between maturity ogives for females when misidentified individuals were included, the *l*_*50*_ and *A*_*50*_ estimates were more disparate than the males.

### Life history of *C*. *amblyrhynchos*

**Fig 4:**
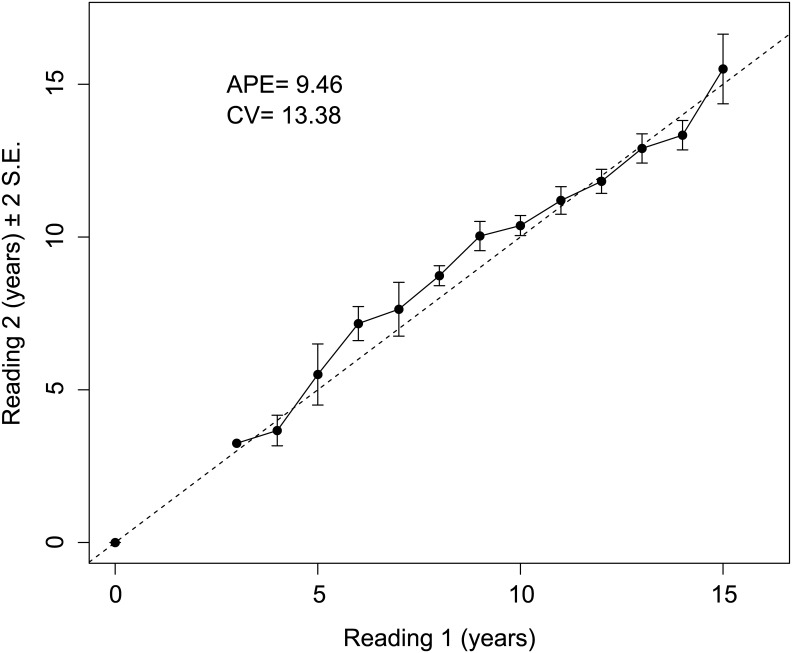
Age-bias plot for *C*. *amblyrhynchos* incorporating the age-specific agreements between Readers 1 and 2. Mean age-specific agreements ± 2 standard errors are plotted along a 1:1 equivalence line.

The confirmed number of *C*. *amblyrhynchos* used in the analyses was 133. This sample consisted of 90 males (71–182 cm TL) and 43 females (102–177 cm TL). The age ranges for males and females were 0–13 and 3–15 years, respectively. The *PA* ± 1 year was 46% with no systematic bias detected by Bowker’s test of symmetry (*df* = 39, *χ*^*2*^ = 43.15, *p* = 0.30). Precision was greatest at younger age classes (< 5 years) (Fig 4). The *APE* and *CV* were 9.46% and 13.38% respectively which are typical for long lived species that have a greater number of growth bands to read [44].

Likelihood ratio tests determined that there was no significant difference between male and female growth curves for any candidate model (VBGF [*df* = 3, *χ*^*2*^ = 1.92, *p* = 0.58]; logistic function [*df* = 3, *χ*^*2*^ = 2.10, *p* = 0.55]; Gompertz function [*df* = 3, *χ*^*2*^ = 2.05, *p* = 0.56]). Therefore, length-at-age estimates were produced with the sexes combined (Fig 2a). All three candidate models produced similar length-at-age estimates that were biologically reasonable; with estimate ranges being *L*_*0*_ = 71–73 cm TL and *L*_*∞*_ = 156–163 cm TL (Table 4). Subsequently, the residual standard error (*RSE*) was similar between all three candidate models and *AICc* determined that they provided equal support for the data (Table 4). Therefore, *MMI* was used to produce model averaged length-at-age estimates (Table 5). The model averaged ${\bar{L}}_{0}$ and ${\bar{L}}_{\infty }$ were 72 cm TL and 159 cm TL respectively (Table 4). Length-at-age estimates for *C*. *amblyrhynchos* from this study (PNG) were similar to estimates from northern Australia [10] (Fig 2c).

**Table 5:** Model averaged total length-at-age estimates for *C*. *amblyrhynchos* over the age range included in this study.

Age	Model averaged TL estimate (cm)
0	72
1	84
2	94
3	104
4	112
5	120
6	126
7	132
8	136
9	140
10	143
11	146
12	148
13	150
14	152
15	153
16	154

Male and female *C*. *amblyrhynchos* mature at different lengths and ages. The maximum likelihood estimates of *l*_*50*_ and *A*_*50*_ predicted for males were 123 cm TL (*SE* = 2.9) and 5.9 years (*SE* = 2.03) respectively (Fig 3a and 3b). Female estimates of *l*_*50*_ and *A*_*50*_ were predicted as 136 cm TL (*SE* = 0.64) and 9.1 years (*SE* = 0.65), respectively, demonstrating that females mature at greater lengths and older ages than males (Fig 3c and 3d).

## Discussion

The misidentification of sharks by observers can have significant effects on the results of life history studies. The inclusion of individuals of species other than *C*. *amblyrhynchos* added substantial error to the life history analyses from growth models. The greatest error was introduced to the growth analysis which produced inaccurate length-at-age and parameter estimates. In contrast, the amount of error introduced to the maturity ogive analysis was marginal relative to the growth analysis, demonstrating that error can be variable between life history parameters. The maturity estimates (*l*_*50*_ and *A*_*50*_) produced for both sexes when misidentified individuals were not removed were similar to those of *C*. *amblyrhynchos*. However, despite producing biologically realistic *l*_*50*_ and *A*_*50*_ estimates, including misidentified individuals produced male maturity ogives that were significantly different from those of *C*. *amblyrhynchos*. These maturity ogives along with the length-at-age estimates would have introduced substantial error to future demographic analyses had species identifications not been verified. Consequently, failing to use accurately identified individuals would have precluded this life history information from being usable due to the obvious magnitude of its error.

Regional variability in growth can occur for carcharhinid species [45]. *Carcharhinus amblyrhynchos* from PNG grows slightly faster than the northern Australian population, although the length-at-birth and the lengths at older ages are similar between the two populations [10]. However, no sexual dimorphism in growth curves occurred for *C*. *amblyrhynchos* in this study nor from northern Australia [10]. Additionally, females matured at greater lengths and older ages than males for both populations, a trait typical of many carcharhinid species [17, 46]. Validation techniques such as marginal increment analysis and mark and recapture were precluded for this study. However, annual growth band deposition is likely based on partial results from validation attempts in northern Australia [10]. In the PNG population, *C*. *amblyrhynchos* were aged to a maximum of 15 years which was younger than in northern Australia (19 years) [10]. This is likely an artefact of the length-dependent mortality of the PNG population by the dome-shaped selectivity of longline fishing. As increased adult mortality prevents individuals from reaching maximum age, these individuals are often rarer in fished populations and are under-represented in stock assessments [47].

This study has shown that substantial error may be introduced when misidentified individuals are unknowingly included in life history analyses. The misidentification rate detected in this study for *C*. *amblyrhynchos* is similar to the largest misidentification rate quantified in the northern Australia observer program [19]. Therefore, this study likely demonstrates the full impact of species misidentification on subsequent life history analyses. The severity of this impact was magnified by the inclusion of misidentified individuals that were far larger and older than verified *C*. *amblyrhynchos* individuals. As growth curves are fitted by minimising the sum of squared residuals, they are strongly influenced by the oldest and youngest data points in the sample [42]. Therefore, the inclusion of two misidentified individuals that had disparate length-at-ages to *C*. *amblyrhynchos* inflated the *L*_*∞*_ estimate of the candidate growth models. As growth parameters co-vary with one another [48] an inflated *L*_*∞*_ estimate also caused an overestimated *L*_*0*_ parameter. The maturity analyses were not influenced as strongly by these misidentifications as sex-specific ogives meant fewer misidentifications were included in each sample. Further as the two largest misidentified individuals were both males, the female maturity ogive was therefore unaffected. Despite minimal error added to the maturity parameters for males, the shape of the ogive was still inaccurate with these misidentifications included. Therefore, the greatest amount of error will be added to life history estimates when misidentified individuals that have length-at-ages which are substantially larger than the true population are incidentally included.

When life history data include outliers, an argument could be made for removing potentially spurious data points. However, removing these individuals from the data without verifying their identity is poor practice. In this study, a *C*. *leucas* individual was identified as *C*. *amblyrhynchos* with a length of 284 cm TL; a value far larger than any other individual in the sample. However, there are confirmed records of *C*. *amblyrhynchos* that were larger than 250 cm TL [49] despite individuals rarely exceeding 190 cm TL [4]. Therefore, removing this large *C*. *leucas* individual from the sample could have potentially removed an individual from an under-represented demographic of the population. In reality *C*. *amblyrhynchos* individuals that reach this maximum size would likely be older than a comparably sized *C*. *leucas* individual. Therefore, a growth curve produced with *c*.250 cm TL *C*. *amblyrhynchos* individuals would not resemble the inaccurate growth curve produced with misidentified individuals in this study. This situation demonstrates that removing supposedly spurious data points should not be a valid option without a reasonable justification.

The recent advancements in genetic techniques means that they are now an important tool in fisheries science [21]. DNA barcoding detected all of the species misidentifications in this study; avoiding the estimation of inaccurate life history parameters. However, the diagnostic images taken by the observers were also an important resource. While they did not detect all of the species misidentifications, the post cruise inspection of images detected the majority of them; including the two outliers that introduced the majority of the error to the growth curve. In a number of instances, some observers took multiple diagnostic images for individuals whose identities were uncertain in order to maximise their identification accuracy. Therefore, providing the observers with cameras not only allowed misidentifications to be detected (in a cost efficient way) but also meant that observers were more vigilant for potential misidentifications. The presence of misidentifications in observer datasets also highlights the need for improved regional species identification guides in many instances, particularly in developing nations.

Genetic analyses are the only option for determining species identifications when poorly resolved images or only parts of an animal (e.g. fin clips or fillets) are available. However, the cost of such an approach means that the incorporation of DNA barcoding into any life history analyses which emanate from observer programs can be cost prohibitive and not always a realistic tool. In contrast, images are a cost effective means for species identifications (particularly from field observations) as long as the image resolution is suitable and the correct lateral view of the animal (with diagnostic features) are taken. Providing observers with cameras so that they can take diagnostic photographs of each specimen (or at least those to be used in subsequent life history analyses) should be considered a feasible addition to observer program sampling methodologies. Such an approach would be especially beneficial for studies that focus on species that are morphologically similar to others and which are likely to be misidentified; genetic validation however still provides the greatest species resolution [19]. By verifying species identifications, accurate data is available to form the basis of life history information and demographic estimates on which informed fishery and population management can be based.
